# Comprehending non-native speakers: theory and evidence for adjustment in manner of processing

**DOI:** 10.3389/fpsyg.2014.01546

**Published:** 2015-01-21

**Authors:** Shiri Lev-Ari

**Affiliations:** ^1^Department of Psychology, University of ChicagoChicago, IL, USA; ^2^Psychology of Language Department, Max Planck Institute for PsycholinguisticsNijmegen, Netherlands

**Keywords:** psycholinguistics, non-native speakers, working memory, comprehension, expectations, top-down processing

## Abstract

Non-native speakers have lower linguistic competence than native speakers, which renders their language less reliable in conveying their intentions. We suggest that expectations of lower competence lead listeners to adapt their manner of processing when they listen to non-native speakers. We propose that listeners use cognitive resources to adjust by increasing their reliance on top-down processes and extracting less information from the language of the non-native speaker. An eye-tracking study supports our proposal by showing that when following instructions by a non-native speaker, listeners make more contextually-induced interpretations. Those with relatively high working memory also increase their reliance on context to anticipate the speaker's upcoming reference, and are less likely to notice lexical errors in the non-native speech, indicating that they take less information from the speaker's language. These results contribute to our understanding of the flexibility in language processing and have implications for interactions between native and non-native speakers.

## Introduction

A major goal of theories of language comprehension is to describe how people understand what speakers mean from what they say. Though rarely stated explicitly, such theories focus exclusively on native speakers of the language. Yet the use of a non-native tongue is highly prevalent. For instance, though English has one of the highest numbers of native speakers, many more people use it as a non-native tongue (Crystal, 2003). Accounts of language comprehension say little about how people comprehend such non-native speakers, or about whether such a theoretical account is needed at all. In general, the language of different speakers varies in the demands it imposes on the listener, as well as in its reliability. While people could potentially treat all input similarly, it might be more beneficial to adjust the way one approaches each source according to the contextual needs and the source's properties. We propose that people indeed make such adjustments when they process the language of non-native speakers. In this paper, we provide an account of how people understand non-native speakers of a language, and examine some of the consequences of such adjustment.

While there are no current models for processing the content of what non-native speakers say (as opposed to adapting to the foreign accent), a-priori, there seem to be three classes of potential theoretical accounts. The first potential account is an “invariance” account that assumes that language processing is independent of the nature of the input. According to such an account, listeners process what non-native speakers say in the same way as they process what native speakers say. If the non-native speaker uses ungrammatical structures, or an inappropriate term, the listener would either misunderstand, or would eventually realize the error, and correct it by taking into account the situation and that the speaker is not a native speaker.

A second potential account, an “intelligibility” account, assumes that because non-native speech is less intelligible, it would influence processing in a manner similar to the way increased noise influences language processing. Adverse conditions that don't allow listeners to encode the sound, often lead listeners to rely on top-down processes to interpret what they hear. For example, when listeners listen to familiar words in which one of the phonemes is replaced with a cough, listeners hear the word as intact, because they restore the missing phoneme using their lexical knowledge (Warren, 1970). Similarly, ability to interpret and learn distorted speech depends on lexical knowledge. Listeners can adapt to distorted speech when they listen to words and know what the lexical target is, but not if they listen to non-words, and therefore cannot rely on higher lexical knowledge (Davis et al., 2005). Adjustment to non-native language in this manner, then, would be perceptually driven. The harder it is for listeners to process the language, the more likely they would be to use a top-down manner of processing.

A third, expectations-based account, assumes that in general, language processing varies with listeners' expectations. In the case of non-native speakers, the theory assumes that the comprehension process itself changes from the outset, due to assumptions about the linguistic competence of non-native speakers. Here we argue for this expectations account, and present evidence that supports it and distinguishes it from the alternative accounts.

To illustrate our theory, imagine that you are buying a brownie at a coffee-shop, and your friend comments that he too likes those *chocolate pies*. You are likely to be confused, perhaps check if there are any pies for sale, or try to recall a mention of pies earlier in the conversation. Yet if your friend is a non-native speaker of English, you might be much less confused, because you will rely on your knowledge that non-native speakers are less competent in their non-native tongue, which renders their language less reliable in conveying their intentions. You might therefore interpret his comment as referring to brownies rather than pies. In general, it is not surprising that knowledge and expectations regarding non-native speakers' lower competence lead listeners to interpret what they say differently. Here we propose that such expectations do not only play a role in the final interpretation of non-native language, but that the expectations lead to a change in the very manner that the language is processed from the outset. Specifically, we argue that when listening to non-native speakers, people increase their reliance on top-down processes and decrease the amount of information they take from the language, often sufficing with less-detailed representations. Therefore, not only are you likely to interpret your friend's comment as referring to brownies, but you might not even notice that your friend referred to brownies with the inappropriate label *chocolate pies*.

Next we will review previous research on the role of top-down processes and less detailed representations in language processing in general. Then we will describe the expectations account regarding processing language of non-native speakers, and detail the ways in which its predictions differ from those of an intelligibility account. Finally, we will describe an experiment that tests the expectations account's prediction and distinguishes it from the intelligibility account.

### Top-down language processing and the role of speaker-induced expectations

Top-down processes are an integral part of language processing. The N400 ERP component, which reflects semantic integration, is modulated by the predictability of the linguistic context (Federmeier and Kutas, 2005). The role of supportive or misleading contexts in facilitating or delaying processing has also been observed behaviorally in reading times (Duffy et al., 1988). Non-linguistic context can also influence language processing. For example, the sentence “Put the apple on the towel in the box” is temporarily ambiguous at the point the listener hears “on the towel,” and the non-linguistic context can influence its interpretation. In the absence of any context, the preferred interpretation of “on the towel” is as the destination of the action. Yet, the presence of a contrast set—an apple on a towel and an apple on a napkin—facilitates the interpretation of “on the towel” as a modifier (Tanenhaus et al., 1995). Similarly, affordances of objects in the visual context can override syntactic processing defaults (Chambers et al., 2004).

Statistical learning also plays an important role in language processing and interpretation (e.g., MacDonald, 1994; Wells et al., 2009; Fine and Jaeger, 2011). These studies show that listeners learn the distribution of different structures in the language of their interlocutor or in the language as a whole, and use this information to adjust the weight they give the different structures in processing. While it is often unclear whether these effects are top-down or bottom-up, and they do not show generalization to reliance on cues beyond the specific learned structures, they do show that past experience can shape processing.

Some evidence indicates that listeners treat unreliable speech differently from reliable speech. Specifically, while in general listeners' phonetic representations are influences by the speech that they hear, this is not the case when the speech is clearly unrepresentative, and therefore, unreliable, as when the speaker holds a pen in her mouth (Kraljic et al., 2008). A particularly interesting and relevant study shows that expectations can influence the weight given to a cue as a whole during lexical access (McQueen and Huettig, 2012). Participants listened to sentences containing a critical word that fit one of four pictures on the screen. One of the remaining three pictures fit a phonetic neighbor, which differs in word onset, and another picture fit a phonetic neighbor which differs in rhyme. The fourth picture was unrelated. In general, neighbors which overlap in their onset compete for lexical access more than neighbors which overlap in their rhyme. In this study, however, a manipulation of the reliability of the audio signal *in filler words* (by adding interference similar to that generated by AM radios), led to decreased reliance on onset information compared to rhyme information. This study thus shows that learning that a cue is less reliable overall, can influence reliance on that cue during processing even when the cue is locally valid in the processed stimuli. Our proposal is similar in nature, but examines whether a less reliable cue can influence processing in contexts where there is no direct evidence that the cue is unreliable, namely, whether the unreliability of the language of non-native speakers affects processing even when they say exactly the same thing as a native speaker. It further examines the role of working memory in modulating the ability to adjust reliance on different cues, and generalizes to a different linguistic level and different cues.

### Speaker-specific expectations

Listeners also hold specific expectations regarding their interlocutors, and these can influence the way they process and interpret language. When listeners hold prior expectations that do not fit the situation, these expectations may influence and even distort the very perception of the speech. Niedzielski (1999) demonstrated that listeners' performance in a vowel-matching task is influenced by their expectations regarding the way residents of certain geographic regions pronounce these vowels. Johnson et al. (1999) demonstrated that information about the gender of the speaker influences listeners' determination of phoneme boundaries in a vowel perception task. Listeners' perception of accent is also influenced by their expectations. Rubin (1992) presented listeners with recorded speech of a native speaker of American English accompanied by a fictitious photo of the speaker. When the photo was of an Asian woman, participants perceived the speech to be more accented than when it was of a Caucasian woman. Expectations, then, can impact the way people judge or evaluate elements of language.

ERP evidence suggests that expectations of the speakers are integrated into the interpretation already at the initial stages of processing. For instance, the voice of the speaker creates expectations related to the gender of the speaker. Content that violates these expectations, such as hearing a male voice saying “If only I looked like Britney Spears in her latest video,” evokes an N400 effect similar in timing and scalp distribution to that elicited by a semantic anomaly (Van Berkum et al., 2008). Foreign accent also raises expectations—ones of lower linguistic competence and greater likelihood of grammatical errors. These expectations are similarly integrated early in the comprehension process. Consequently, while a grammatical error that is committed by a native speaker evokes a P600 component, it does not evoke it when committed by a non-native speaker. (Hanulikova et al., 2012).

In line with this neurocognitive evidence are findings that show that expectations can influence not only the interpretation of language but also the way it is processed. For example, expectations can override habitually inferred implicatures. For instance, disfluency in naming an object typically indicates that the object is difficult to name. Consequently, when a speaker is disfluent, listeners infer that the speaker is trying to refer to a difficult-to-label-object rather than to an easy-to-label object. However, if the listeners believe that the speaker suffers from “object agnosia,” then disfluency no longer leads to such inference (Arnold et al., 2007). Similarly, when a speaker says “a tall cup,” listeners infer the presence of more than one cup. But listeners do not infer such a contrast set from the use of a modifier if the speaker's use of modifiers is unreliable (Grodner and Sedivy, 2014). The process of re-mapping phonemic categories according to the acoustic information in order to deal with the variability in speech, a process known as “Talker Normalization,” is also influenced by listeners' expectations. Thus, listeners remap the phonemic categories when they believe that the variability in speech is due to a change of speaker, but not when they believe that the variability is within a talker (Magnuson and Nusbaum, 2007).

### Less-detailed representations

Listeners and readers sometimes do not process language in full, but only to a level that is “good enough” for the purpose at hand (e.g., Christianson et al., 2001, 2010; Ferreira et al., 2001, 2002; Ferreira, 2003; Ferreira and Patson, 2007). In the same spirit, people do not always incorporate the full details of a word meaning into the representation of an utterance, but they make do with the content that is essential for their task (Sanford, 2002; Sturt et al., 2004; Sanford et al., 2005). For example, readers are more likely to notice changes in text that involve elements that are in linguistic focus than elements not in focus, presumably because focal elements are most relevant (Sturt et al., 2004). Similarly, when not necessary, people do not always resolve the reference of anaphors (Klin et al., 2006). Such less-detailed representations are often guided by a plausibility heuristic. That is, world knowledge guides the processing and might lead to a representation that is inconsistent with the syntactic structure but is consistent with general knowledge. For instance, sentences such as “The dog was bitten by the man” are sometimes understood to mean that the dog bit the man, which is more plausible (Ferreira et al., 2002). Together, these studies suggest that the level of detail, or even accuracy, of the representation of utterances varies with context. Both less detailed processing and expectation-guided processing are central to the theory we present next about how people comprehend non-native speakers.

### Listening to non-native speakers: the expectations account

Non-native speakers' linguistic competence is typically inferior to that of native speakers (Ellis, 1985). Non-native speakers may make grammatical errors, and may use constructions that native speakers do not use. In addition, their choice of words is often suboptimal and might even be incorrect. As a result, the language of non-native speakers is less reliable in conveying their intention than the language of native speakers. Therefore, it is adaptive for listeners to reduce their reliance on what non-native speakers say, and instead increase their reliance on contextual and other top-down information and top-down processes, compared to when they listen to native speakers. Needless to say, there are great individual differences in how proficient different non-native speakers are, and the degree of adaptation that listeners make is likely to depend on their knowledge and expectations of the non-native speaker's proficiency. In this paper, we focus on processing the language of an unknown non-native speaker that one expects to have lower proficiency that a native speaker.

The processing of language produced by non-native speakers can therefore be characterized by (a) extraction of less information from the language itself and (b) greater reliance on the context and on top-down processes in general. Our proposal is that when listeners process the language of non-native speakers, they devote more resources to top-down processes, including processes aimed at predicting what the non-native speaker is about to say, at the expense of devoting resources to specifying the details of the linguistic input, unless the situation requires them to. Additionally, listeners modify the weight they give to different sources of information. They increase the weight they give to extra-linguistic information and decrease the weight given to linguistic information. When in conflict, then, extra-linguistic information is weighted more heavily. In terms of temporal sequence, listeners start with the context, their knowledge and their expectations, and use them to guide the processing of the language of a non-native speaker. Initially, they process the language at a broad and less detailed level. For example, the listener's representation for the reference *pie* would be broader if it is said by a non-native rather than a native speaker, and could apply even to instances such as a brownie, which a native speaker would not consider to be a pie. Context or general knowledge is then used to specify the meaning. For example, if the visual context includes a baked dessert, the representation would be specified in accordance with it. If such information is not available, the listener might suffice with the broader, less detailed representation of the utterance. Only if the situation requires a more detailed representation, as when there is a need to distinguish between referents, would the listener proceed to process the language more fully.

One implication of the expectations account is that the final representation of the language of non-native speakers is often less detailed than the final representation of the language of native speakers. This is because listeners rely on the context to specify the language, yet the context often does not provide sufficient information about the subtleties of meaning. Initial evidence suggests that this is indeed the case. Listeners are less likely to notice changes in peripheral details in stories told by a native vs. a non-native speaker. Importantly, the reduced amount of detail in the representation is not due to reduced intelligibility as it only occurs when listeners listen for comprehension. When attempting to memorize the sentences, listeners are equally sensitive to changes in details with native and non-native speakers (Lev-Ari and Keysar, 2012).

In general, working memory determines people's ability to control their attention. Working memory resources also constrain the types of information one can use when processing language (Just and Carpenter, 1992; Federmeier and Kutas, 2005; Traxler et al., 2005). For example, individuals with higher working memory are better able to use the animacy of a noun in order to adjust their interpretation of the syntactic structure of the sentence. Thus, the inanimacy of the first noun in a sentence, such as: “The evidence examined by…” facilitates its processing compared with the processing of the temporally ambiguous sentence: “The defendant examined by…” for individuals with higher working memory capacity, but not for those with low working memory capacity (Just and Carpenter, 1992). This is argued to be due to the fact that individuals with higher working memory capacity are better able to integrate the animacy information quickly and adjust their processing accordingly. As the verb *examined* requires an animate subject, they immediately assign *evidence* the object role, and correctly interpret the phrase as passive. Similarly, the animacy of a noun has been shown to minimize the relative difficulty of processing object-extracted relative clauses compared with subject-extracted relative clauses, but again, only for individuals with higher working memory capacity (Traxler et al., 2005). Previous research, then, indicates that the ability to integrate cues of different types and use them to adjust processing manner requires working memory resources. Furthermore, adjustment of the manner of processing requires flexibility in focusing attention and integration of multiple types of information. Therefore, working memory should influence listeners' ability to adjust their manner of processing. In particular, individuals with high working memory should have a greater ability to integrate their expectations regarding speakers' linguistic competence, and use it to adjust their processing.

The invariance account differs from the expectations account in all predictions. It argues that listeners will process the same information similarly, regardless of who the speaker is, and therefore will not differ in the amount of information they will extract from the linguistic input or in their reliance on top-down information. In contrast, the intelligibility account shares some of the predictions of the expectations account. When speech is less intelligible, listeners increase their reliance on top-down processes to restore degraded input (e.g., Warren, 1970). Accented speech is less intelligible, and this alone could encourage greater reliance on top-down processes. Though this prediction parallels that of the expectations account, the two accounts differ in a crucial way. They differ in the reasoning behind the filtering of the linguistic input, as well as in their prediction regarding the influence of working memory resources on the adjustment of manner of processing. The expectations account proposes that listeners process the language of non-native speakers in less detail because they expect non-native language to be less reliable. They therefore devote their resources to higher levels of processing, namely to predictive processes and to greater attention and reliance on contextual information. The expectations account assumes that the integration of speaker expectations is an effortful process that requires attentional resources and flexibility in controlling attention. Therefore, it predicts that the higher listeners' working memory is, the better able they would be to adjust to the speakers. In contrast, an intelligibility account that is based on performance with degraded input predicts that the perceptual difficulty alone would lead listeners to divert their resources from bottom-up processing to top-down processing. This should occur at all levels of working memory. Alternatively, if low working memory prevents even input-driven adjustment, the performance of listeners with low working memory should be poorer when processing non-native language, since the input is less intelligible, and they exhibit greater detriments in performance when the task is more difficult. In either case, an intelligibility account, then, would predict that even at low levels of working memory, listeners should perform differently with native and non-native speaker.

Our study tests the expectations account's proposals that language processing is guided by context to a greater degree when listening to non-native speakers, that the representation of non-native language is less detailed, and that the ability to adjust to non-native speakers is constrained by cognitive resources.

## Experiment

To evaluate the predictions of our theory, we tracked the eye movements of participants while they followed the instructions of either a native or a non-native speaker. To test the proposal that people rely on context more when they listen to non-native than to native speakers, we induced contextual expectations by telling participants that the speaker will instruct them to click on a series of pictures of objects that share a theme. We did not tell them what the theme was. For example, for one set of pictures participants first followed instructions to click on “the witch,” “the man on the magic carpet,” and “Santa.” The implied contextual theme was imaginary creatures. This created the expectation that the next object will be the remaining imaginary creature, in this case, a mermaid. To the extent that the contextual theme guides processing, listeners should be fixating on the mermaid already at word onset, just before they hear the name of the next item. If the expectations account is correct, then such anticipation should be more pronounced when the speaker is a non-native speaker, reflecting a comprehension process that is more context-guided.

In addition to evaluating listeners' reliance on prior expectations, we were able to assess how such expectations impacted the process of reference assignment. The speaker did not say “mermaid.” Instead she instructed the participant to click on the *f* ε*ri*. The intended target was a ferry, which is thematically related to the previous objects via the less dominant theme of “means of transportation,” as Santa was riding a sleigh, the witch was riding a broom, and the man was on a magic carpet. Yet *f* ε*ri* also sounds like fairy, so if participants expect the next item to be an imaginary figure, and if participants sometimes engage in less-detailed processing, they might process *f* ε*ri* shallowly enough that it might even include a mermaid. When they search for a “fairy,” then, they may accept the mermaid as the intended referent, without even noticing the discrepancy between the label “fairy” and the competitor mermaid. If our account is correct, then people should be less likely to consider the ferry as the referent, and more likely to choose the picture of the competitor, mermaid, when they are instructed by a non-native than by a native speaker. In addition, if we are correct, then such adjustment to a non-native speaker should be more manifest when participants have a higher working memory capacity.

### Method

#### Participants

*Eighty-five* undergraduate students participated in the study for either credit or payment. All were native speakers of English. One participant was excluded because of a computer error.

#### Stimuli preparation and pretesting

We constructed 10 sets, each containing three context-building pictures that shared two themes, and two additional pictures, each sharing only one of these themes. One of the themes was more dominant than the other. For example, the witch, the man on the magic carpet and Santa shared the dominant theme of imaginary creatures, as well as the secondary theme of using a means of transportation. Each trial started with the instructions to click on three pictures, which elicited the expectation that instructions to click on a fourth contextually related picture (mermaid) would follow. Yet these critical instructions always included a homophone (e.g., *fεri*), which fit a semantically related object that was not present (e.g., fairy) as well as the actual target whose thematic relation to the other three pictures is less salient (e.g., ferry)^1^. In addition, there were three filler pictures in each trial that were unrelated to the critical reference, the other pictures or the themes (e.g., scissors). Locations of the target, competitor, context building pictures and filler pictures varied from set to set.

Stimuli were pre-tested to ensure that they induced the dominant theme. A different group of participants received a booklet, such that each page contained a grid with eight pictures. The right column of the grid contained the three pictures that were supposed to elicit expectations about a theme. Participants were instructed to examine the other five pictures and circle the one that has something in common with the three pictures on the right. This checked whether the three pictures were most readily grouped with the competitor. After participants finished the booklet, they were asked to review it again, and to choose a different picture for each set that also shares something with the three pictures. If they did not identify the picture that fit the secondary theme, the experimenter pointed to the target picture and asked whether they could name a property that all four pictures shared. This second pass checked whether a relation between the three pictures and the target picture could be found. Participants then received a different booklet with pictures of the competitor and target objects as well as filler objects. Their task was to decide whether each object could be referred to with a given label. This confirmed that while the critical labels were appropriate for the target objects, they were inappropriate for the competitor objects. For example, it confirmed that “ferry” is appropriate for the ferry but that “fairy” is an inappropriate name for the mermaid.

Based on the pre-test, we selected 10 sets. For each set, the competitor was selected by participants at least 80% of the time during the first pass, and the target was selected or was recovered at least 80% of the time. The labels were always judged as acceptable for the target pictures and as unacceptable for the competitor pictures. This ensured that the competitor pictures are more contextually appropriate but do not fit the labels, whereas the target pictures could fit the context, but to a lesser degree, yet they fit the labels well. We used 17 additional sets as fillers.

Instructions were recorded in English by two females, one native speaker of English and one native speaker of Mandarin. We chose Mandarin because Chinese students are the largest group of non-native speakers on campus. The critical instructions were always of the form “Click the [target word].” The instructions for the filler and context-building items were of similar form, but sometimes included words such as *next* or *now* in the beginning of the phrase. The two speakers were recorded together in an attempt to equate the speech as much as possible. For each instruction, the native speaker was recorded first, and then the non-native speaker attempted to imitate her speech rate and intonation. Indeed, the total length of the instructions phrases did not differ between the two speakers [*t*_(9)_ = 1.2, *p* > 0.05]. Target word onset for the critical items, which was calculated as the duration between the beginning of the utterance and the beginning of the first phoneme of the target word, was almost identical for the two speakers (*M* = 710 and 713 ms for the native and non-native speaker, respectively). We selected the non-native speaker, because while her accent was clearly detectable, it was still easy to understand her. The recordings started with the speaker explaining the task. To create the impression that the non-native speaker has low linguistic competence in English, she made errors that are typical of non-native speakers of English whose native tongue is Mandarin, such as “There have” instead of “There are” (Yip, 1995; Chan, 2004). The non-native speaker did not make such errors with the critical instructions, and the native speaker did not make such errors at all. The instructions made it clear to the participant that the speaker was looking at the same pictures, but that some of the pictures were numbered for the speaker. The speaker's task was to instruct the participant to click on each picture in the given order. The participants, then, was led to believe that while the experimenter determined only the identity and order of the pictures, and that the speaker determined how to name them.

#### Procedure

Participants were told that they will perform two tasks. First, they performed a verbal working memory task (Unsworth et al., 2005). Participants determined whether sentences were sensible and received a letter for memorization after each sentence. After each set of sentences and letters, which ranged in length from three to seven, participants were asked to recall the letters they had memorized in the order they received them. Participants' working memory score was determined by the number and length of full sets they recalled. Then, they were told that they would participate in a communication task which involved eye-tracking.

Participants sat in front of a computer with a Tobii T120 eye-tracker. They were told that their task is to follow instructions recorded by a previous participant to click on pictures that share a theme. The experimenter first calibrated the eye-tracker, and then played the introductory part of the instructions. Before the experiment started, the participants were allowed to ask the experimenter clarification questions. The entire session took about 40 min.

### Data analysis

In order to test the hypotheses of the expectations account, it is crucial that the language and the contextual expectations are pitted against one another. The items were pre-tested in an offline paradigm that evaluated how objects are grouped, but not whether context-building objects actually create expectations. We wanted to make sure that the items lead the participants to expect that the competitor, rather than the target, would be referred to after the three context-building objects. Therefore, after the first 12 participants, we examined whether participants looked longer and more often at the competitor than they did at the target *prior* to word onset, as an index of contextual expectations. For three of the items, there was no such competitor advantage in either number of fixations or fixation duration. This means that we could not use those items to test our hypothesis. Therefore, those three items were removed from all analyses and were not coded for any additional participants.

In all analyses, we included only trials in which the participant selected either the target or the competitor in response to the critical instructions. This was the case for all but six trials (1%), in which participants selected the competitor by error before the critical instructions. These trials were excluded from all analyses.

In all analyses that included Working Memory as a factor, we used Working Memory as a continuous variable. Participants' Working Memory scores ranged from 3 to 75 (possible scores: 0–75), with a mean of 48, a median of 50, and a standard deviation of 16.4. For follow up analyses aimed at examining the interaction between Working Memory and other variables, we grouped participants into High and Low Working Memory groups, and analyzed each group separately. Participants who scored up to 50, the median score, on the Working Memory task were classified as having Low Working Memory (*N* = 43), and those who scored over 50 on the Working Memory task were classified as having High Working Memory (*N* = 41)^2^.

### Results and discussion

#### The product of comprehension: picture selection

**Figure 1:**
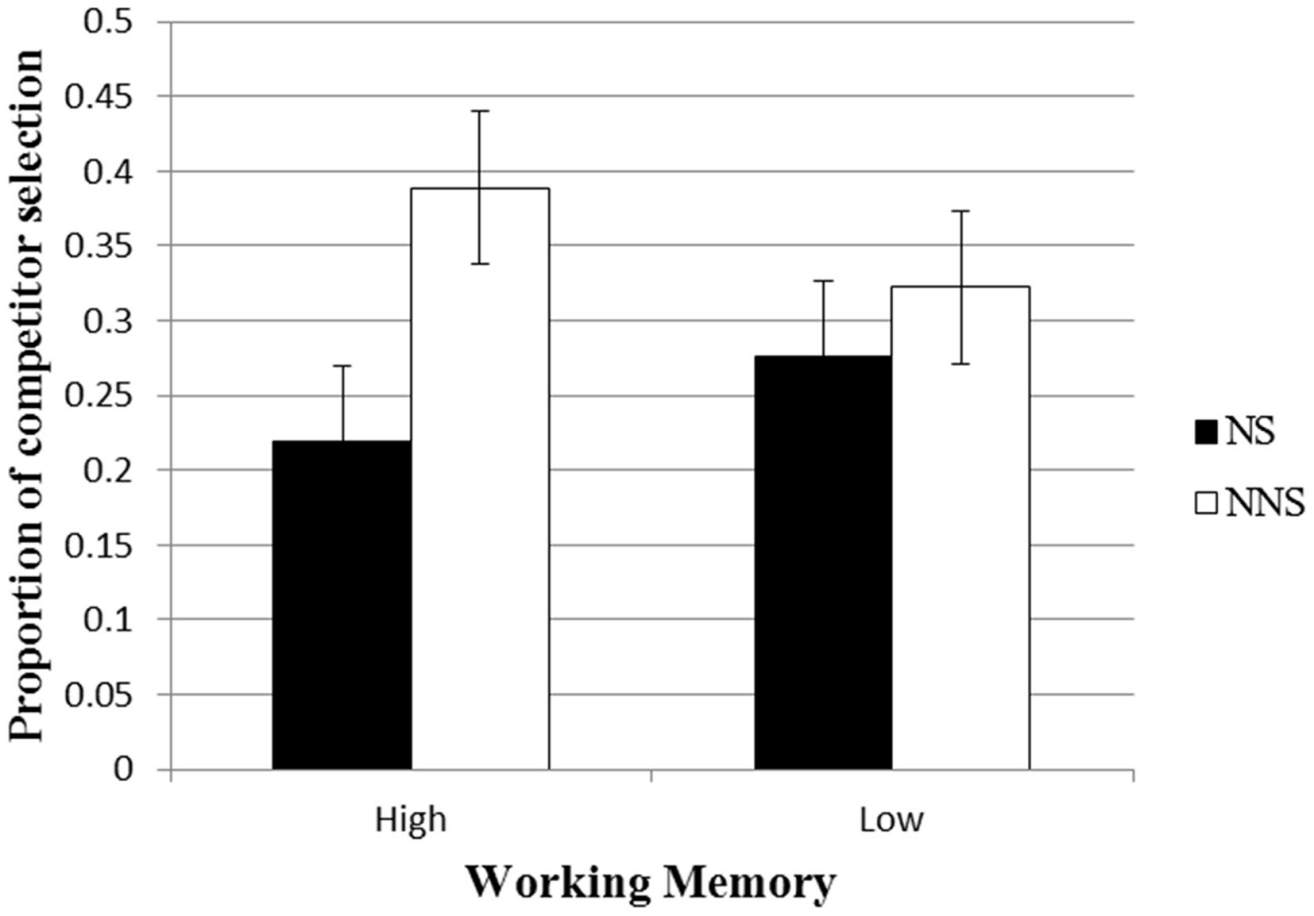
**Proportion of competitor selection as a function of Speaker and Working Memory**.

First, we tested whether the selection of the referent was influenced by the speaker being non-native, and whether this interacted with Working Memory. Choosing the competitor (mermaid) would suggest an impact of the dominant context, while choosing the target (ferry) would suggest a bottom-up interpretation of the language. As predicted, participants were more likely to select the competitor with a non-native speaker than a native speaker (Means = 36% and 25%, respectively). A logit analysis with Subjects and Items as random variables and Working Memory, Speaker and their interaction as fixed variables revealed a main effect of Speaker (β = 0.66, *Z* = 2.18, *p* < 0.03)^3^. The difference between those following instructions made by native vs. non-native speakers was numerically larger for participants with High Working Memory (22% vs. 39%) than for those with Low (28% vs. 32%), but the interaction was not significant (*Z* < 1) (See Figure 1, and Table A in Appendix B in Supplementary Material for the full results table). This pattern shows that when people interpret what a non-native speaker says, they are more likely to rely on context even in their final choice of referent. It also suggests that people with High Working Memory might be better able to adjust in this way to the speaker, but this is statistically inconclusive.

The results are inconsistent with the invariance account, which predicts no difference between the way that listeners would interpret the language of native and non-native speakers. In contrast, they are consistent with the expectations account which predicts greater reliance on context in interpreting language due to expectations of reduced reliability of the language itself. The intelligibility account, in contrast, would predict this finding, but for different reasons. It assumes that difficulty with processing the speech leads to greater reliance on context. If difficulty induced the effect, then one might expect that participants who listened to non-native speakers would take longer to respond than those who listened to native speakers. In fact, participants who listened to the native speaker took on average 76 ms longer to select a picture than the participants who listened to the non-native speaker, although this difference was not significant. One might still argue that listeners did not take longer with the non-native speaker because intelligibility led them to not fully process the speech, and just rely on their expectations. If so, they might not even notice the discrepancy and could even be faster than participants who listened to the native speaker. We will next proceed to examine why participants reached a different interpretation with a non-native speaker. These results would also help further distinguish between the intelligibility and the expectations accounts.

#### The process of comprehension: reliance on context

**Figure 2:**
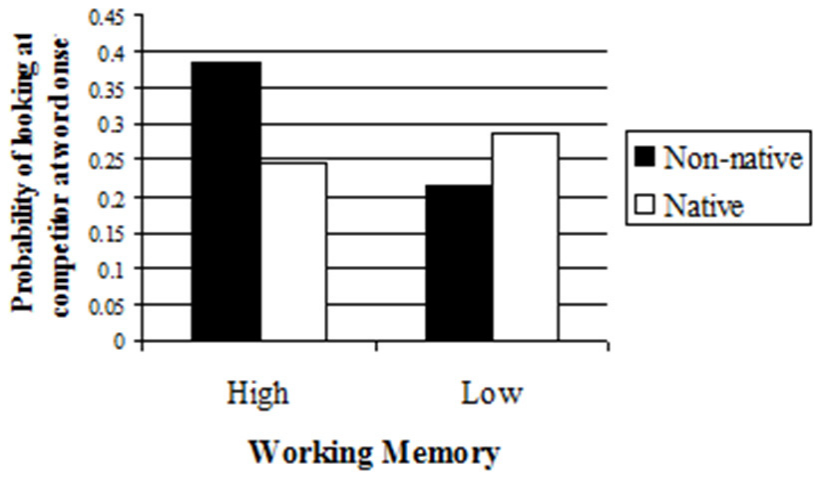
**Probability of looking at the competitor at word onset as a function of Speaker and Working Memory**.

We examined whether participants are more likely to rely on context to guide their interpretation when listening to non-native speakers, and whether Working Memory moderates this effect. To test reliance on context, we examined the likelihood that participants would use the theme suggested by the context to anticipate and guide processing. We considered it a case of anticipation if the listener was already looking at or started to look at the contextually-appropriate competitor at word onset. For each trial of each participant, we examined whether participants were fixating on the competitor at the exact moment at which the onset of the critical word occurred^4^. As we predicted, participants were more likely to look at the competitor already at word onset when listening to a non-native speaker than a native speaker but only if their Working Memory was high (See Figure 2). A logit with Items and Subjects as random variables and Working Memory and Speaker as fixed variables revealed a marginal effect of Working Memory (β = 0.02, *Z* = 1.795, *p* < 0.08) and the predicted Working Memory X Speaker interaction (β = −0.03, *Z* = −2.04, *p* < 0.05. See Table B in Appendix B in Supplementary Material for full results table). To examine the nature of the interaction, we ran separate logit analyses on High and Low Working Memory participants. A logit analysis with Subjects and Items as random variables and Speaker as a fixed variable on the group of participants with High Working Memory revealed a main effect of Speaker (β = −0.66, *Z* = −2.39, *p* < 0.02). Participants were more likely to look at the competitor at word onset when listening to a non-native speaker than a native speaker (Means = 0.41 and 0.24, respectively). A similar logit analysis on the group of participants with Low Working Memory did not reveal any significant differences (Means = 0.25 and 0.325, respectively; *Z* = 1.07, *p* > 0.2).

These results indicate that participants with high working memory adapt to the speaker just as the expectations account predicts: they rely on the context to a greater degree when listening to non-native speakers, and use it to anticipate the speaker's words. In contrast, participants with low working memory, who have fewer resources to make such an adjustment, do not adapt in this way to the speaker. These results, similarly to the picture selection results, are incompatible with an invariance account that would predict similar behavior when listening to native and non-native speakers. Furthermore, the role that working memory plays in modulating the ability to adjust the manner of processing to the speaker shows that the adjustment is not due to difficulty of processing the speech. Had difficulty been the cause, working memory would have played no role or would have led to the opposite pattern, showing greater difference for low working memory participants, because they should have had even greater difficulty processing non-native speech. Instead, participants with low working memory perform similarly with native and non-native speakers. The findings that low working memory participants perform similarly with both native and non-native speakers, indicates that processing difficulty is not the trigger for adjustment. In fact, it is those with high working memory that adjust to the non-native speaker and rely more on context, and this supports the claim that the adjustment is driven by ability to take into account expectations about the speaker.

#### The process of comprehension: target advantage

Patterns of anticipation show that participants with High Working Memory are more likely to use the context with a non-native than with a native speaker. They do this in order to anticipate what the speaker would say and to ultimately select an object in line with the context. Here we consider how much they attend to the content of the language itself. The expectations account predicts that when listening to a non-native speaker, participants may be less likely to notice the discrepancy between the label (e.g., *f* ε*ri*) and the object they wrongly select (e.g., mermaid). To test the extent that listeners notice the discrepancy and consider the literal referent (e.g., ferry), we examined whether the target was fixated on preferentially, compared to a filler. We call this measure Target Advantage. We calculated the Target Advantage by averaging the number of fixations on the filler pictures in each trial between word onset and the response, and subtracting it from the number of fixations on the target in that trial. For example, if a participant fixated twice on the target, once on each of two filler pictures, and never on the third filler, the Target Advantage score would be 1.33 (2–2/3).

**Figure 3:**
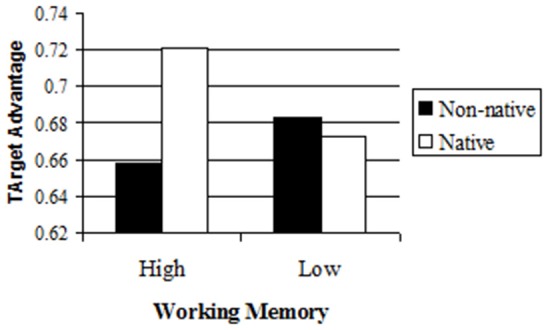
**Target Advantage as a function of Speaker and Working Memory**. Target Advantage is the difference between the number of fixations on the target and the number of fixations on the average filler.

We analyzed the data with a mixed model. The model included Items and Subjects as random variables and Speaker, Working Memory and their interaction as fixed variables. The results showed a marginal effect of Speaker (β = 0.14, *t* = 1.91) such that participants had a marginally larger Target Advantage with a native speaker than with a non-native speaker (Means = 0.85 and 0.7, respectively). More importantly, there was an interaction between Speaker and Working Memory (*t* = 2.88, See Table C in Appendix B in Supplementary Material for the full table of results)^5^. In order to examine the nature of the interaction, we conducted separate mixed model analyses on the groups of participants with Low and High Working Memory. A mixed model with Subjects and Items as random variables and Speaker as a fixed variable for the group of participants with High Working Memory showed an effect of Speaker (β = 0.29, *t* = 2.71), such that participants showed a larger Target Advantage with the native speaker than with the non-native speaker (Means = 0.95 and 0.65, respectively). Participants with high working memory differed in their number of fixations on the target with the native vs. the non-native speaker (1.75 vs. 1.41, respectively; *t* = 2.48), but not in their number of fixations on the average filler picture (0.81 vs. 0.76, respectively, *t* < 1)^6^. In contrast, for participants with Low Working Memory there was no effect of Speaker (β = −0.05, *t* < 1, Means = 0.72 and 0.77, respectively; See Figure 3). They did not differ in the number of fixations on the target when listening to native vs. the non-native speaker (1.46 vs. 1.5, respectively; *t* < 1), nor did they differ in the number of fixations on the average filler picture (0.75 vs. 0.73; *t* < 1).

These results suggest that participants with High Working Memory were less likely to consider the target as a referent when listening to a non-native speaker than when listening to a native speaker whereas participants with Low Working Memory considered the target as a referent to the same degree with the two speakers. This suggests that participants with High Working Memory were less likely to notice the discrepancy between the label and the competitor with the non-native speaker. On the other hand, participants with Low Working Memory were equally likely to notice the discrepancy and look at the target with the native speaker and the non-native speaker.

The pattern of results for Target Advantage supports our account but it is possible that we did not find a difference in the Target Advantage for the Low Working Memory group for a different reason. Perhaps the Low Working Memory participants had greater difficulty deciding between the two pictures when listening to the non-native speaker. This could have induced longer deliberation, which could in turn increase the number of fixations on the target relative to the fillers, thereby increasing the Target Advantage. But if this were true, then it should have also led to an increase in fixations on the competitor relative to fillers, or to a “Competitor Advantage.” To test whether this was the case, we conducted a similar mixed model analysis on Competitor Advantage. A model with Subjects and Items as random variables and Speaker, Working Memory and their interaction as fixed factors did not reveal any effects (all *t*'s < 1). Therefore, the pattern of Target Advantage could not have been due to a general increase in fixations on the target and competitor.

These results suggest that listeners with high working memory are more likely to engage in less-detailed processing when listening to non-native speakers. Therefore, they are less likely to notice the discrepancy between the language and the context. In contrast, listeners with low working memory seem to be less likely to adjust to non-native language. These results cannot be explained by the intelligibility account. Research on processing degraded input shows greater reliance on top-down processes (e.g., Davis et al., 2005), but it doesn't predict this when the input is intelligible enough to be processed. The fact that participants with low working memory did not process non-native language in less detail relative to native language shows that it was intelligible enough for them. If the speech was intelligible enough for participants with low working memory, it certainly was so for those with high working memory. Therefore, it is unlikely that participants with higher working memory switched to top-down processing with non-native language due to degradation of the speech. These results are of course also incompatible with the invariance account, as, again, they show a difference in performance when listening to non-native vs. native speakers.

Together, the results indicate that listeners interpret the language of native speakers and non-native speakers differently, as well as that they reach these interpretations via a different manner of processing. When listeners process non-native language, they increase their reliance on contextual information and use it to guide their processing, and occasionally allow it to override linguistic information. In addition, they extract less information from the language and therefore are less likely to notice the discrepancy between the language and the appropriate label for the context. The adjustments, however, are dependent on cognitive resources, and therefore the higher a listener's working memory is, the more likely the listener is to adjust to the speaker. This pattern of results shows that the adjustment is not simply due to greater difficulty in processing non-native language. The greater adjustment by those with higher cognitive resources, especially when that led to an impoverished representation in cases when a detailed one could be created with the input, is better explained by an account that assumes that adjustment is driven by an attempt to optimize processing according to expectations than by an account that proposes that it is driven by difficulty of processing the input.

Because working memory capacity varied naturally in the experiment, it is possible that our results are due to a factor that correlates with working memory. This possibility is always present with such natural variation. One reason to believe that working memory itself is responsible for our results is that the pattern we find is analogous to other results with working memory in the literature. For example, low working memory individuals fail to integrate contextual information in online tasks, but do integrate it to perform similarly to people with high working memory on off-line tasks (Dagerman et al., 2006; Madden and Zwaan, 2006). Similarly, while working memory in our experiment impacted the on-line processing, it did not affect the final representation. Participants were in general more likely to select the competitor (mermaid) with a non-native speaker, and that did not significantly interact with working memory. Therefore, it might be the case that working memory influences listeners' ability to adjust their manner of processing from the outset, allowing expectations to influence what they attend to, but that eventually listeners use their expectation to modify their interpretation regardless of working memory. This would suggest that listeners with high and low working memory do not differ in their expectations regarding non-native speakers but only in how quickly they can use them.

## General discussion

The meaning of what people say is context dependent. Therefore, constructing meaning from linguistic input is a process that requires integration of many types of information and relies on both bottom-up and top-down processes. There is growing evidence that during language processing people integrate different types of information, both linguistic and non-linguistic. Our account focuses on the role of expectations that listeners have of the speaker. It proposes that these expectations shape the manner in which meaning is constructed by influencing which types of information are used and at what point in time they are integrated. The study we presented focuses on the case of interactions between a native speaker and a non-native speaker. They show that listeners' expectations of a non-native speaker lead them to rely more on top-down information, such that they weigh context more heavily to guide the interpretation process and determine the final interpretation. The study also demonstrates that listeners' expectations of a non-native speaker lead to the filtering of some of the linguistic information, leaving the listeners with coarser, less-detailed representations.

Such flexibility in the manner of language processing might allow listeners to optimize processing. While it is valuable to integrate many types of information, it might also be valuable to adjust the reliance on those types of information according to their reliability. There are always multiple types of information that could be used when processing language, such as linguistic properties and language statistics, visual context, general knowledge, information about the preferences and opinions of the speakers or information about their linguistic competence or level of knowledge on different issues (e.g., Just and Carpenter, 1992; Tanenhaus et al., 1995; Sturt et al., 2004; Arnold et al., 2007; Clayards et al., 2008; Hanulikova et al., 2012). Integrating all these types of information might be costly, inefficient and, if irrelevant, might even lead the listener astray. Therefore, it is possible that selecting between different types of information and adjusting their weight and the manner in which they are used might be important for successful and efficient communication. Understanding such flexibility in the language processing mechanism would allow us to better understand the comprehension process, why some people are better at it than others, and in which situations those differences would be enhanced or ameliorated.

One factor that influences reliance on different cues is their availability. For example, low proficiency, as in the case of second language learners, reduces the ability to rely on semantic context quickly enough to guide prediction of upcoming lexical items (Martin et al., 2013). Our study highlights another factor that modulates individuals' ability to integrate different cues—individuals' general cognitive resources, such as working memory. Previous research has already shown that working memory can influence ability to quickly integrate different types of information during language processing and thus influence processing at both the semantic (Federmeier and Kutas, 2005) and grammatical levels (Just and Carpenter, 1992; Traxler et al., 2005). Our study extends these findings to the integration of non-linguistic information. While our study focused on processing of lexical items, working memory is likely to modulate the influence of the nativeness of the speaker on processing at other linguistic levels as well. The reliability of the language of non-native speakers is reduced not only in terms of lexical choice, but also at the grammatical, phonological, and even pragmatic level. Therefore, individuals with higher working memory capacity should increase reliance on non-linguistic cues and reduce their reliance on linguistic cues at all linguistic levels when processing the language of non-native speakers.

This study focused on the case of processing the language of non-native speakers as a case of processing language when there are specific expectations of the speaker. Listeners are likely to have expectations of many other types of speakers, such as children, the elderly and so forth, and are likely to adjust their processing to optimize communication according to their specific expectations regarding linguistic ability, cognitive ability and so on in each case.

Our study evaluated how listeners adjust the way they process non-native language. Our findings demonstrate that listeners are flexible, but they also raise questions about the extent and limitation of such flexibility. For example, do listeners adjust similarly to all non-native speakers, driven by general expectation for reduced competence or is the adjustment sensitive to the perceived level of linguistic competence of the specific interlocutor? Can listeners adjust the manner of processing dynamically as more information about the speaker's competence becomes available? On the one hand, listeners' demonstrated flexibility suggests they might be able to dynamically adapt. On the other hand, the very adjustment to less-detailed processing might not allow them to process the language deeply enough to dynamically evaluate its reliability, since they may not even notice the errors that the speaker makes.

### Listening to non-native speakers and the role of cognitive load and intelligibility

Processing accented speech imposes a cognitive load (Munro and Derwing, 1995), and when input is degraded, people increase their reliance on top-down processes (Warren, 1970). Yet we show that the impact of expectations on processing is different from that of cognitive load and lower intelligibility. In general, research that examined whether cognitive load leads to less-detailed representations finds only reduction in performance, but no influence of load on the granularity of the representation (Sanford et al., 2005). In this section, we evaluate whether a cognitive load account or an intelligibility account can provide an alternative explanation for our results. We show that while some of our findings are consistent with a cognitive load account or an intelligibility account, the majority of the findings are not.

In general, lower intelligibility could encourage top-down processing, and thus could have led to higher selection of the competitor and to higher likelihood of looking at the competitor at word onset. Yet reduced intelligibility should predict either greater shift to top-down processing by those having the greatest difficulty, or simply poorer performance by those who are having the greatest difficulty. In contrast, our results indicate that it is higher working memory that leads to greater deviation between performance with native and non-native speakers. As it is unlikely that individuals with higher working memory had greater difficulty than individuals with lower working memory in understanding the speech, it is unlikely that reduced intelligibility alone is responsible for the pattern we found.

One may hypothesize a hybrid intelligibility account that argues that adaptation is input-driven yet effortful. Such an account could predict adjustment of processing by individuals with higher working memory but not by individuals with low working memory. While this account cannot be ruled out completely for this study, this account would need to explain why lower intelligibility leads to less-detailed representations when the speech is intelligible enough to be processed in full. After all, the participants with low working memory performed the task well, indicating they were able to process the language in full. Another piece of evidence supporting the interpretation that the greater adjustment that individuals with higher working memory exhibit is due to expectations rather than intelligibility comes from a previous study mentioned earlier, that shows adjustment to non-native speakers takes place only when expectations are relevant for the task at hand. Thus, when individuals listen for comprehension, and therefore the meaning matters, they adjust their processing to non-native speakers. When individuals are asked to listen with the goal of memorizing the speech, and therefore the reliability of the lexical choice is irrelevant, individuals no longer adjust their processing manner (Lev-Ari and Keysar, 2012). An intelligibility account should predict an adjustment of processing in both cases, as the intelligibility of the speech remains the same. It seems more economical to assume that adjustment is driven by the same mechanism in both studies rather than that it is driven by some form of intelligibility in one study, and by communicative expectations in another.

### Further implications

The role that working memory plays in modulating one's ability to adjust to speakers extends previous studies that demonstrate that working memory can influence language processing by constraining one's ability to make timely use of different types of information (Just and Carpenter, 1992; Federmeier and Kutas, 2005; Traxler et al., 2005). Our studies demonstrate that the higher one's working memory, the more that person adjusts to non-native speakers. Yet working memory resources might also change with circumstances, and so the same person might adjust to different degrees under different circumstances. Therefore, if adjustment contributes to the success of the interaction, then some situations or types of interactions that occur under circumstances that drain the resources that are necessary for adjustment might lend themselves to greater communicative difficulties. Another potential consequence is that the ease and success of communication between native and non-native speakers might change with life trajectory. The older people get, the fewer cognitive resources they have available (e.g., Wingfield et al., 1994), reducing the ability to adjust and possibly hindering communication with non-native speakers.

The ability to adjust to non-native speakers, and to speakers in general, is especially interesting because of its potential social implications. Differences in ability to adjust could influence the smoothness of the interaction, and therefore the affective evaluation of the interlocutors and the desire for further interaction. Furthermore, the difference in the amount of encoded details can also influence interlocutors' impression of the speaker. For example, it seems likely that differences in encoding of subtleties of the message can influence agreement with the speaker. Yet our findings suggest that if the interlocutor is a non-native speaker, listeners will be less likely to react differently to subtleties of the message, especially if they have more available cognitive resources.

The differential attention to the context might also have implications for interpersonal judgment. For example, people tend to take behavior as indicative of people's intention and disposition, and to under-weigh the impact of the situation on the behavior (Jones and Harris, 1967; Ross, 1977). But if people are more tuned to context when they listen to non-native speakers, they might be able to take the situation into account more effectively with non-native speakers than with native speakers. If this is true, it would indicate that manner of language processing has a wide-ranging effect, allowing a cognitive mechanism which is designed to optimize the comprehension process influences our social interaction in important ways.

This paper makes a novel contribution to theories of language comprehension and communication. It describes how listeners adjust to non-natives' lower competence by allowing their expectations to guide the very processes of comprehension. It shows that these expectations affect the final interpretation and the amount of detail in the representation. Before your non-native friend even says anything, you are already using context more intensely to anticipate what she would say. She talks about a “chocolate pie” even when there are no pies around, but you are not confused. Your interpretation of “pie” is more general, leading you to a chocolate cake, without even noticing the discrepancy. Technically, it is an error on the part of the speaker and the listener, as “pie” is not an appropriate reference to a chocolate cake. However, from the standpoint of the processing system, this might be more optimal.

### Conflict of interest statement

The author declares that the research was conducted in the absence of any commercial or financial relationships that could be construed as a potential conflict of interest.

## Footnotes

^1^The speakers who recorded the stimuli received a script with the target words written in accordance with the spelling of the target object (e.g., ferry). The pronunciation of all critical pairs is homophonous in the U.S. Midwest, where the experiment was conducted.

^2^The groups slightly differ in size because there were a few people who received the median score, and we opted to classify people with the same WM score at the same WM level.

^3^In all analyses in this experiment, the model included the intercepts for the random variables, but did not include slopes. For each and every analysis, we checked whether adding slopes was necessary but in all cases the slopes either did not improve the model when models with and without slopes were compared with a Likelihood Ratio Test (Baayen, 2008, p.275), or led to overparameterization and therefore dropped (Jaeger, 2008).

^4^Since the eye-tracker we used samples participants' eye-gaze every 8 ms, the point in time at which we coded participants' eye-gaze might have been up to 7 ms away from word onset.

^5^Note that the dependent measure in the analyses of Target Advantage was continuous rather than binary. R does not report p -values in such a case, as there is no consensus regarding the calculation of degrees of freedom. The common method is to interpret as significant t-values of 1.96 and above, as in the normal distribution.

^6^Subtracting the number of fixations on the average filler from the number of fixations on the target might not lead to the exact number we report in the Target Advantage analysis, because these are all predictions of the model that we ran rather than raw fixation numbers. Since we ran three different models: one for the Target Advantage, one for the number of fixations on the target and one for the number of fixations on the fillers, the clustering of the random variables might differ a little between the models, leading to slightly different results.
