# Corrigendum: Comprehending non-native speakers: theory and evidence for adjustment in manner of processing

**DOI:** 10.3389/fpsyg.2015.00574

**Published:** 2015-05-06

**Authors:** Shiri Lev-Ari

**Affiliations:** Psychology of Language, Max Planck Institute for PsycholinguisticsNijmegen, Netherlands

**Keywords:** corrigendum, psycholinguistics, non-native speakers, working memory, comprehension, expectations, top-down processing

The Acknowledgment Section in the original publication was incomplete. The full acknowledgment is:

The research was funded by National Science Foundation grant BCS-0849034 awarded to principal investigator Dr. Boaz Keysar, who also provided guidance in this project. We would like to thank Chloe Goldman for helping with running the studies and Sayuri Hayakawa for helpful comments on an earlier version of the manuscript.

## Conflict of interest statement

The author declares that the research was conducted in the absence of any commercial or financial relationships that could be construed as a potential conflict of interest.

