# Supplementary material for: Comprehending non-native speakers: theory and evidence for adjustment in manner of processing
Source: Front Psychol. 2015 Jan 21;5:1546. doi: 10.3389/fpsyg.2014.01546 (PMC4300909; doi:10.3389/fpsyg.2014.01546)
Supplement: Supplementary file 1 [file DataSheet1.DOCX]

Appendix A: Experimental stimuli used in Experiment 1

| **Critical word** | **Context building items** | **Implied theme** | **True theme** | **Competitor** | **Target** | **Included?** |
| --- | --- | --- | --- | --- | --- | --- |
| Bat | wooden eagle (“eagle”), wooden horse (“horse”), wooden dolphin (“dolphin”) | Animals | Wooden objects | Black bird | Baseball bat | Yes |
| Ring | bracelet (“bracelet”), necklace (“necklace”), round earrings (“earrings”) | Jewelry | Round things | Brooch | Rubber ring | No |
| Steak/stake | pig roasting on a stick (“roasting pig”), corn dog (“corn dog”),  kebab (“kebab”) | Meat | (Things on) a stick | Sliced ham | Stake | Yes |
| Fairy/Ferry | witch on a broom (“witch”), man on a magic carpet (“man on magic carpet”), Santa with his sleigh (“Santa”) | Fantasy | Modes of transportation | Mermaid | Ferry | Yes |
| Flower/Flour | broccoli (“broccoli”), lettuce (“lettuce”), herbs (“herbs”) | Vegetation | Food | Plant | Flour | Yes |
| Glasses | glass saltshaker (“saltshaker”), glass plate (“plate”),  glass bottle | Kitchen items | Glass items | Bowls | Eye-glasses | No |
| Pie/Pi | round cookie (“cookie”), round piece of cheese (“cheese”), tortilla (“tortilla”) | Food | Circle | A rectangular piece of chocolate cake | π | Yes |
| Tape | syringe (“syringe”), blood pressure monitor (“blood pressure monitor”), thermometer (“thermometer”) | Health | Measurement | Bandage | Measuring tape | No |
| Chips | stacked slices of cheese (“cheese”), kebab (“kebab”), multilayered sandwich (“sandwich”) | Food | Stacked items | Crackers | Poker chips | Yes |
| Bow | slingshot (“slingshot”), noose (“noose”), nunchuk (“nunchuk”) | Weapons | Things that have a string | Arrow | Ribbon | Yes |

Appendix B: Result tables of statistical analysis

A. Results table for Competitor selection in Experiment 1

| Random effects:  Variable name Variance Std.Dev.  Subject 1.03413 1.01692  Item 0.57707 0.75965  Number of obs: 581, groups: Subject, 84; Item, 7  Fixed effects:  Estimate Std. Error z value Pr(>\|z\|)  (Intercept) 0.791765 0.356840 2.219 0.0265 *  Speaker 0.656878 0.301652 2.178 0.0294 *  Working Memory -0.008248 0.013180 -0.626 0.5315  Speaker:Working Memory 0.014408 0.018428 0.782 0.4343 |
| --- |

B. Results table for Fixations on competitor at word onset in Experiment 1

| Random effects:  Variable Name Variance Std.Dev.  Subject 0.315288 0.56151  Item 0.051254 0.22639  Number of obs: 588, groups: Subject, 84; Item, 7  Fixed effects:  Estimate Std. Error z value Pr(>\|z\|)  (Intercept) -0.83173 0.17993 -4.623 3.79e-06 ***  Speaker -0.21427 0.22375 -0.958 0.3382  Working Memory 0.01818 0.01013 1.795 0.0727 .  Speaker:Working Memory -0.02819 0.01382 -2.040 0.0413 * |
| --- |

C. Results table for Target Advantage in Experiment 1

| Random effects:  Variable name Variance Std.Dev.  Subject 0.012859 0.11340  Item 0.125097 0.35369  Residual 0.738761 0.85951  Number of obs: 580, groups: Subject, 84; Item, 7  Fixed effects:  Estimate Std. Error t value  (Intercept) 0.710029 0.144240 4.923  Speaker 0.144482 0.075799 1.906  Working Memory -0.004432 0.003420 -1.296  Speaker:Working Memory 0.013529 0.004702 2.877 |
| --- |
